# InSe‐Enabled Wearable Triboelectric Nanogenerator for Biomechanical Energy Harvesting and Motion Sensing

**DOI:** 10.1002/open.70272

**Published:** 2026-08-04

**Authors:** Sisi Wang, Yongwei Huang

**Affiliations:** ^1^ School of Physics and Materials Science Guangzhou University Guangzhou China; ^2^ School of Physics Ningxia University Yinchuan China

**Keywords:** human motion monitoring, InSe, triboelectric nanogenerators (TENGs), wearable sensor

## Abstract

Self‐powered wearable sensors are highly desirable for continuous human motion monitoring, yet the development of flexible devices with high output performance and good environmental adaptability remains challenging. Herein, an InSe‐based triboelectric nanogenerator (IS‐TENG) is developed through a simple layer‐by‐layer assembly strategy using Kapton as the flexible substrate, aluminum foil as the electrode, PTFE as one triboelectric layer, and compacted InSe powders as the counterpart friction material. Benefiting from the effective interfacial contact and favorable charge‐generation capability of the PTFE/InSe triboelectric pair, the fabricated IS‐TENG delivers a high open‐circuit voltage (*V*_OC_) of 773 V, a short‐circuit current (*I*_SC_) of 35 μA, a transferred charge (*Q*_SC_) of 229 nC, and a maximum output power of 465 μW. The device also exhibits reliable capacitor‐charging capability, good cycling durability, and stable output under varying operating and environmental conditions. More importantly, it can function as a wearable self‐powered sensor for finger tapping detection, motion‐state recognition, and joint bending monitoring. These results demonstrate the promise of the IS‐TENG for biomechanical energy harvesting and intelligent wearable motion sensing.

## Graphical abstract

An InSe‐enabled wearable triboelectric nanogenerator is developed using a PTFE/InSe friction pair for biomechanical energy harvesting and self‐powered motion sensing. The compacted InSe layer promotes interfacial contact and charge generation, enabling high electrical output, capacitor charging, durable operation, and wearable detection of finger tapping, motion states, and joint bending.

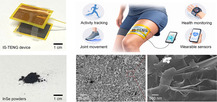

## 1 Introduction

Human motion monitoring has emerged as a critical component in a wide range of fields, including healthcare [1], athletic training [2], rehabilitation [3], and intelligent safety management [4]. Reliable tracking of body movements is of great significance because it enables continuous evaluation of how the human body responds to external stimuli and mechanical demands in real time [5, 6]. In sports‐related scenarios, motion data can be used to analyze movement regularity, posture control, force transmission, and technical execution, thereby providing useful guidance for training optimization and injury reduction [7, 8, 9, 10, 11, 12, 13]. In clinical rehabilitation, continuous monitoring of joint motion, limb coordination, and recovery status offers a more objective basis for assessing therapeutic progress and tailoring treatment plans for individuals with motor dysfunction [14, 15, 16, 17, 18]. In everyday health applications, motion monitoring also provides valuable information for estimating activity level, identifying signs of physical fatigue, and improving exercise quality and safety [19, 20]. This function is especially important in aging societies, where long‐term observation of gait behavior, posture stability, and mobility changes may contribute to the early warning of movement disorders and fall‐related risks in elderly populations [21, 22, 23]. Meanwhile, the rapid expansion of wearable systems and artificial intelligence has raised expectations for next‐generation motion sensors, which are now expected not only to capture physical movement but also to support real‐time feedback, intelligent interpretation, and data‐assisted decision‐making [24, 25]. These growing demands have accelerated the development of wearable sensing platforms that are soft, lightweight, highly sensitive, and suitable for prolonged on‐body operation [26]. As a result, human motion monitoring is gradually shifting from simple motion recording toward a more comprehensive technological framework for health evaluation, behavior analysis, risk prevention, and human–machine interfacing [27, 28, 29, 30].

Traditional motion‐detection systems have long been based on wired instruments and power‐dependent electronic modules [14, 17]. While these platforms are capable of delivering accurate signals in controlled laboratory settings, their practical deployment is often hindered by rigid configurations, poor portability, and limited comfort during on‐body use [18]. These drawbacks make them less suitable for continuous monitoring under daily‐life conditions [31]. Recently, the emergence of flexible electronics has promoted the development of wearable motion sensors with softer structures, lower weight, and improved conformability to human skin [32, 33, 34]. Even so, the majority of currently reported wearable systems still rely on batteries as the primary energy source, and many of their sensing units operate through resistance‐ or capacitance‐based mechanisms that require sustained electrical input [35]. Such dependence not only increases system complexity but also restricts long‐term use, distributed deployment, and maintenance‐free operation [17, 36, 37]. Against this background, triboelectric nanogenerator (TENG)‐based self‐powered sensors have become an appealing solution, as they can harvest biomechanical energy from human motion and simultaneously generate electrical signals for sensing without persistent external power [38, 39, 40, 41, 42, 43, 44, 45, 46, 47, 48, 49, 50, 51, 52, 53]. Owing to this dual functionality, TENG devices have been widely explored for applications such as gait recognition, joint‐motion detection, respiration monitoring, limb movement tracking, and posture assessment [54]. Meanwhile, the triboelectric output of these systems is closely related to the properties of the selected friction materials [55]. Recent studies on material‐based TENGs, including 2D materials, crystalline porous materials, polymer materials, and oxide materials, have demonstrated that rational triboelectric material selection and interfacial engineering play decisive roles in regulating charge generation, charge retention, and output performance [56,57]. Although graphene, MXene, and MoS_2_ have been widely used in TENGs, InSe offers a distinct layered III–VI semiconductor structure with abundant surface sites and favorable interfacial electronic properties, enabling effective contact, charge generation, and charge transfer with polytetrafluoroethylene (PTFE) [58, 59, 60]. In this context, the novelty of the present work lies in introducing InSe as a layered III–VI semiconductor friction material to construct a PTFE/InSe‐based wearable TENG, in which the compacted InSe powder layer provides a rough continuous interface and favorable interfacial electronic characteristics for high‐output biomechanical energy harvesting and self‐powered human motion sensing [61,62]. By introducing InSe as a functional layered semiconductor triboelectric layer, the PTFE/InSe‐based wearable TENG benefits from its van der Waals structure, abundant surface sites, and favorable interfacial electronic properties, enabling enhanced contact, charge generation, and charge transfer for high‐output biomechanical energy harvesting and self‐powered motion sensing. Therefore, exploring new functional materials is highly important for improving charge density, interfacial interaction, and signal stability. Recently, topological materials have drawn increasing attention in electronics and energy‐related fields due to their unique electronic characteristics, stable surface states, and tunable interface behaviors [63]. These advantages indicate that incorporating topological materials into TENG systems may provide a new route toward higher‐performance self‐powered sensors and broaden their potential in advanced wearable human motion monitoring. Besides, topological‐material‐ and chalcogenide‐material‐based TENGs, such as the reported VS_4_‐based TENG, have further demonstrated that materials with unique electronic structures and tunable interfacial properties can provide new opportunities for improving triboelectric charge generation and output performance [64].

In this work, we report an InSe‐based TENG (IS‐TENG) for simultaneous biomechanical energy harvesting and self‐powered human motion monitoring. The device was constructed through a simple and scalable layer‐by‐layer strategy using Kapton as the flexible substrate, aluminum foil as the conductive electrode, PTFE as one triboelectric layer, and compacted InSe powders as the counterpart friction material. Owing to the favorable interfacial contact characteristics and effective charge‐generation capability of the PTFE/InSe triboelectric pair, the fabricated IS‐TENG exhibited excellent electrical output performance, delivering a high open‐circuit voltage (*V*_OC_) of 773 V, a short‐circuit current (*I*_SC_) of 35 μA, a transferred charge (*Q*_SC_) of 229 nC, and a maximum output power of 465 μW. In addition, the device showed stable and tunable output responses under different operating frequencies, separation distances, and applied forces, together with good environmental adaptability under varying humidity and temperature conditions. The IS‐TENG also demonstrated effective capacitor‐charging behavior and maintained stable electrical output after 36,000 operation cycles, confirming its reliable durability and energy‐harvesting capability. More importantly, the device could be directly employed as a wearable self‐powered sensor for monitoring finger tapping, distinguishing representative human motion states such as walking, running, and jumping, and tracking the bending behaviors of the wrist, finger, elbow, and knee at different angles. These results indicate that the proposed IS‐TENG not only serves as an efficient self‐powered energy harvester but also provides a feasible route for developing flexible, lightweight, and low‐power wearable sensing platforms based on advanced functional materials for intelligent motion monitoring and healthcare‐related applications.

## 2 Experiments

### 2.1 Materials

PTFE film, used as one triboelectric layer, was purchased from Shandong Jianuo Engineering Materials Co., Ltd, China. Indium selenide (InSe) powders, employed as the counterpart triboelectric material, were obtained from Beijing Zhongke Xinnuo New Material Technology Co., Ltd, China. Aluminum foil, serving as the conductive electrode, was purchased from Henan Mingtai Aluminum Industry Co., Ltd, China. Kapton film, used as the flexible supporting substrate, was obtained from Beijing Zhixin Network Energy Technology Co., Ltd, China. Electrical wires and other auxiliary materials used for device fabrication and electrical connection were purchased from commercial suppliers. Unless otherwise specified, all materials were used as received without further purification or treatment.

### 2.2 The Fabrication Process of IS‐TENG Device

The IS‐TENG device was fabricated via a facile and scalable layer‐by‐layer assembly route. Initially, Kapton films were cut into the desired dimensions and employed as flexible supporting substrates for the two triboelectric components. To construct the negative triboelectric layer, aluminum foil was first attached onto the Kapton substrate as the conductive electrode, followed by the lamination of a PTFE film onto the aluminum surface, thereby forming a Kapton/aluminum/PTFE trilayer structure. This assembled layer served as one friction interface of the IS‐TENG. For the counterpart friction layer, aluminum foil was similarly fixed onto another Kapton substrate to function as the current collector. Subsequently, InSe powders were uniformly deposited onto the aluminum surface. The IS‐TENG had an effective size of 3 × 2 cm, corresponding to a contact area of 6 cm^2^. The Kapton substrate, aluminum foil electrode, and PTFE film had thicknesses of 50, 30, and 50 μm, respectively. The InSe powders were loaded at approximately 8 mg·cm^−2^ and pressed at 1 MPa for 60 s, repeated three times, to obtain a compact InSe layer. To enhance the interfacial compactness, surface uniformity, and structural stability of the powder coating, the deposited InSe layer was repeatedly pressed until a dense and continuous triboelectric surface was formed, yielding a Kapton/aluminum/InSe configuration. This pressing process was essential for improving the adhesion of the InSe powders and maintaining the integrity of the active layer during repeated contact‐separation cycles. After the preparation of the two functional layers, they were assembled in a face‐to‐face configuration, in which the PTFE film and InSe powder layer acted as the opposing triboelectric surfaces. Finally, conductive wires were connected to the aluminum electrodes to enable electrical output and subsequent performance characterization. Owing to the simplicity of the lamination and pressing procedures, the entire fabrication process is straightforward, low‐cost, and readily adaptable for large‐area and scalable device preparation.

### 2.3 Characterization and Measurements

The electrical performance of the IS‐TENG device was evaluated under well‐controlled experimental conditions. The *V*_OC_ signals were collected using an oscilloscope, while the *I*_SC_ and *Q*_SC_ were measured by an electrometer. To achieve periodic and reproducible contact‐separation motion, the device was mounted on a mechanical excitation system capable of providing programmable actuation. During the measurement process, the working parameters, such as the applied force, separation distance, and operating frequency, were carefully regulated to ensure consistent mechanical stimulation. Besides, ambient conditions were maintained as stable as possible throughout the experiments, and environmental factors such as temperature and relative humidity were monitored to minimize their potential influence on the triboelectric output. All measurements were conducted under the same testing protocol to ensure the comparability and reliability of the obtained data. Under these conditions, the IS‐TENG device exhibited stable electrical signals with good repeatability, providing a solid experimental basis for the analysis of its output behavior, energy harvesting capability, and self‐powered sensing performance.

## 3 Results and Discussion

### 3.1 Design, Fabrication, and Morphology of the IS‐TENG

**FIGURE 1:**
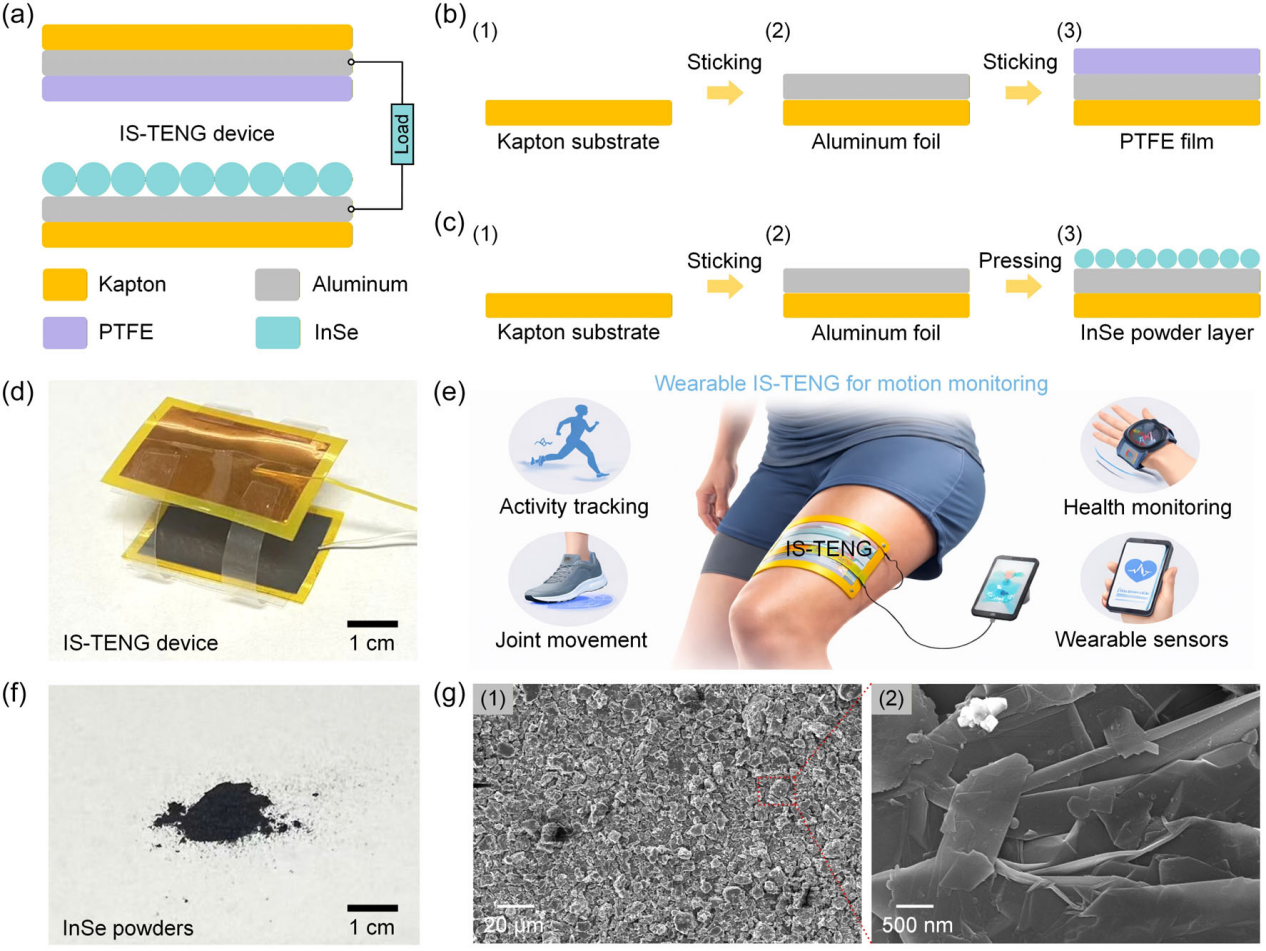
Structural design, fabrication process, practical application, and morphological characterization of the IS‐TENG device. (a) Schematic illustration of the layer‐by‐layer device architecture of the IS‐TENG, consisting of Kapton substrates, aluminum electrodes, a PTFE film, and a compacted InSe powder layer. (b1–b3) Step‐by‐step fabrication process of the PTFE‐side layer: preparation of the Kapton substrate, attachment of the aluminum foil electrode, and lamination of the PTFE film to form the Kapton/aluminum/PTFE structure. (c1–c3) Step‐by‐step fabrication process of the InSe‐side layer: preparation of the Kapton substrate, attachment of the aluminum foil electrode, and deposition/pressing of InSe powders to form the Kapton/aluminum/InSe structure. (d) Photograph of the as‐fabricated IS‐TENG device with scale information. (e) Schematic illustration of the wearable IS‐TENG for motion monitoring. (f) Optical image of the InSe powders used for device fabrication. (g1,g2) SEM images of the compacted InSe powder layer at different magnifications.

Figure 1a shows the structural configuration of the IS‐TENG device. The device consists of two oppositely arranged triboelectric layers operating in a contact‐separation mode. The upper friction layer is composed of a Kapton substrate, an aluminum electrode, and a PTFE film, whereas the lower friction layer includes a Kapton substrate, an aluminum electrode, and an InSe powder layer. Both electrodes are connected to an external load, indicating the electrical output path during operation. This configuration demonstrates that PTFE and InSe act as the two triboelectric materials, while Kapton and aluminum serve as the supporting substrate and current‐collecting electrode, respectively. Figure 1b1 illustrates the first fabrication step of the PTFE‐based triboelectric layer. A Kapton film was first prepared as the flexible substrate. This substrate provides mechanical support for the upper layer and also ensures the flexibility of the final device, which is essential for potential wearable applications. Figure 1b2 presents the second fabrication step for the PTFE side. An aluminum foil was attached onto the Kapton substrate through a simple sticking process. In this structure, the aluminum foil functions as the conductive electrode, which is responsible for charge collection and electrical signal output during device operation. Figure 1b3 shows the final preparation step of the PTFE‐side layer. A PTFE film was further laminated onto the aluminum foil, thereby forming a Kapton/aluminum/PTFE trilayer structure. This assembled layer serves as one triboelectric interface of the IS‐TENG and provides the highly electronegative surface required for triboelectric charge generation. Figure 1c1 displays the initial step in constructing the InSe‐based triboelectric layer. Similar to the PTFE side, a Kapton film was first selected as the flexible supporting substrate. The use of the same substrate material on both sides contributes to structural consistency and facilitates device assembly. Figure 1c2 depicts the second fabrication step for the InSe side, in which an aluminum foil was fixed onto the Kapton substrate. As in the PTFE‐side structure, the aluminum layer acts as the electrode and provides a conductive pathway for electron transfer and signal collection. Figure 1c3 shows the final fabrication process of the InSe‐side layer. InSe powders were distributed onto the aluminum surface and then compacted by pressing to form an InSe powder layer. This pressing treatment is important for improving the compactness and adhesion of the deposited particles, thereby enabling the formation of a relatively stable and continuous triboelectric interface. In contrast to the laminated PTFE film, the InSe layer was constructed through a powder‐deposition and pressing strategy, highlighting the distinct preparation route of this active surface. Figure 1d presents a photograph of the fabricated IS‐TENG device. The image clearly confirms the successful assembly of the two triboelectric layers in a face‐to‐face configuration. The electrical leads connected to the electrodes indicate that the device is ready for output measurement and performance evaluation. In addition, the scale bar of 1 cm reveals the compact size of the fabricated device, which is beneficial for integration into flexible and wearable systems. Figure 1e demonstrates a representative application scenario of the IS‐TENG as a wearable motion‐monitoring device. The sensor is attached to the knee joint, where repeated bending and stretching motions can induce periodic contact and separation between the two triboelectric layers. The surrounding illustrations further indicate its potential applications in activity tracking, joint movement detection, health monitoring, and wearable sensing. This result suggests that the IS‐TENG possesses promising applicability in real‐time human motion monitoring. Figure 1f shows the optical image of the InSe powders used in the fabrication of the device. The dark powder morphology can be directly observed at the macroscopic scale. This image provides a straightforward view of the raw material state before its integration into the triboelectric layer. Figure 1g1 presents the low‐magnification SEM image of the InSe powder layer. A rough and densely packed surface morphology can be clearly observed, indicating that the deposited InSe particles form a textured interface. Such surface roughness is favorable for increasing the effective contact area between the two triboelectric materials, which is expected to enhance charge generation during repeated contact‐separation cycles. Figure 1g2 shows the enlarged SEM image of the selected region in Figure 1g1. A typical layered and flake‐like microstructure can be observed, revealing the characteristic morphology of InSe after deposition. The stacked nanosheet‐like features and irregular edges contribute to the micro/nanostructured surface of the friction layer, which may promote stronger interfacial interaction and improved triboelectric output performance.

### 3.2 Working Mechanism, Flexibility, and Electrical Output Performance of the IS‐TENG

**FIGURE 2:**
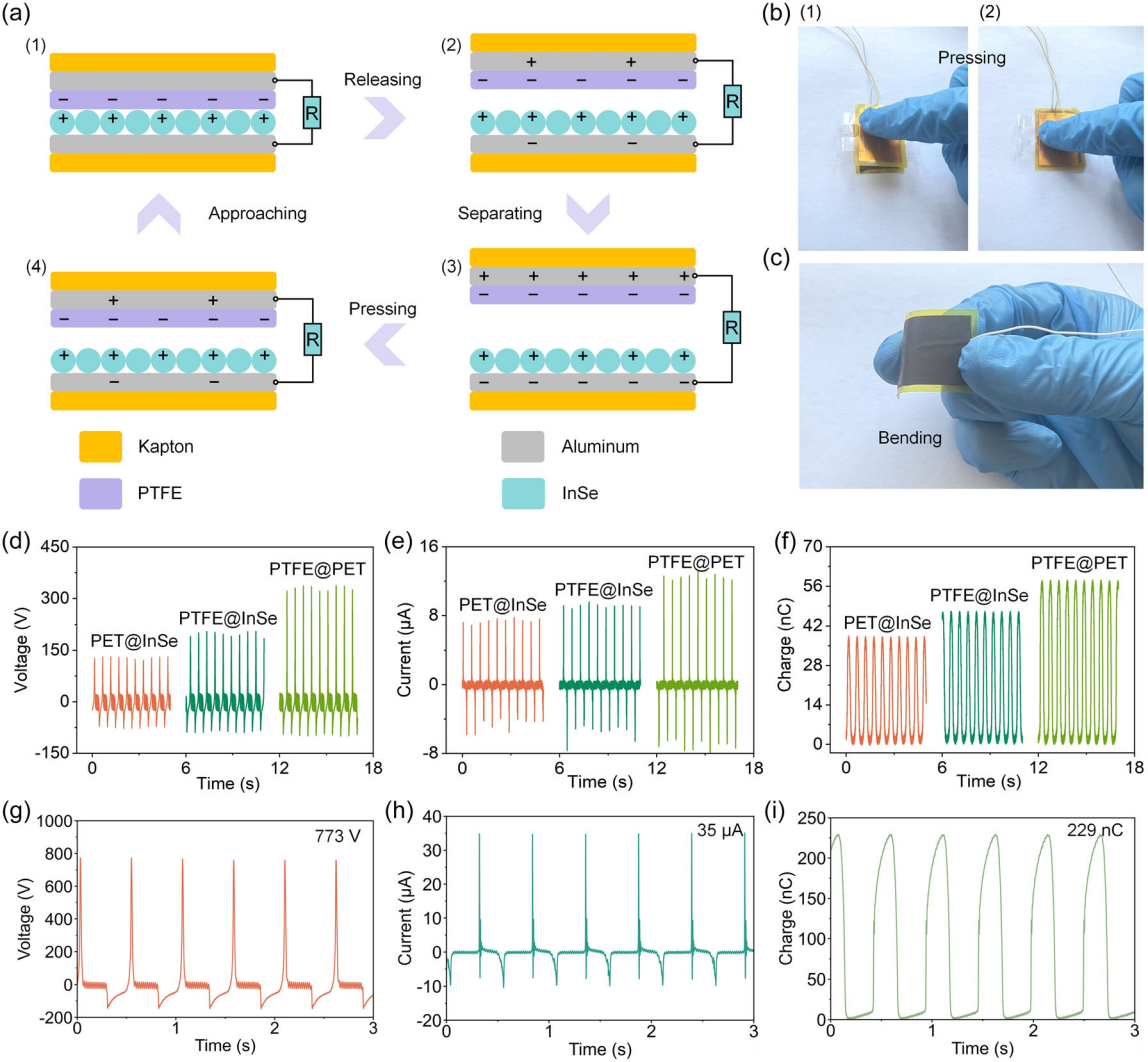
Working principle, flexibility, triboelectric polarity verification, and electrical output characteristics of the IS‐TENG device. (a1–a4) Schematic diagrams illustrating the charge distribution, electron‐flow direction, and contact‐separation working mechanism of the IS‐TENG during a complete operating cycle, including contact, releasing, separated, and approaching states. (b) Optical photographs showing the pressing process of the IS‐TENG under external mechanical actuation. (c) Optical photograph of the bent IS‐TENG, demonstrating its flexibility. (d–f) Output voltage, current, and transferred charge of different triboelectric pairs, including PET@InSe, PTFE@InSe, and PTFE@PET, for verifying the relative triboelectric polarity of InSe. (g–i) Output voltage, current, and transferred charge of the PTFE/InSe‐based IS‐TENG, respectively.

Figure 2a1 illustrates the initial contact state of the IS‐TENG under an externally applied pressing force. In this configuration, the PTFE layer and the InSe friction layer are brought into intimate contact, enabling triboelectric charge generation at the interface. Owing to their different electron affinities, opposite charges are formed on the two triboelectric surfaces after contact electrification. The PTFE side carries negative charges, while the InSe side exhibits positive charges. At this stage, because the two charged surfaces remain in close proximity, no obvious potential difference is established across the external circuit. Figure 2a2 shows the charge distribution and electrical state when the external force is released and the two triboelectric layers begin to separate. As the interfacial distance increases, an electric potential difference is generated between the top and bottom electrodes due to electrostatic induction. This potential difference drives electrons to flow through the external load, thereby producing an electrical output signal. The schematic clearly indicates the redistribution of charges and the corresponding electrical response during the releasing process. Figure 2a3 depicts the fully separated state of the IS‐TENG. In this condition, the PTFE layer and InSe layer are spatially apart, leading to a relatively large electric potential difference between the two electrodes. The separated configuration represents the state in which the induced charge transfer approaches its maximum level within one operating cycle. This stage is essential for achieving effective output generation in the contact‐separation mode TENG. Figure 2a4 presents the approaching process when the two triboelectric layers move back toward each other under renewed external pressing. As the gap distance gradually decreases, the electrostatic field between the two electrodes changes accordingly, forcing electrons to flow back through the external circuit in the opposite direction. This process gives rise to a reversed current signal and completes one full electrical output cycle of the IS‐TENG. Figure 2b1 shows a practical photograph of the IS‐TENG under finger pressing. The image demonstrates that the device can be directly actuated by an external mechanical stimulus without the need for a complicated driving setup. This result visually confirms the feasibility of manually triggering the contact process of the device and reflects its potential for self‐powered sensing under real mechanical interactions. Figure 2b2 further displays the pressed state of the device during manual operation. Compared with the previous image, this panel provides additional visual evidence of the structural response of the IS‐TENG under compressive deformation. The photograph suggests that the device maintains structural integrity during pressing, which is important for ensuring stable electrical output during repeated use. Figure 2c presents the bending behavior of the IS‐TENG. The device can be easily bent without noticeable structural failure, indicating its excellent flexibility and mechanical adaptability. Such a flexible characteristic is highly desirable for wearable and deformable electronics, since it allows the IS‐TENG to conform to curved surfaces and dynamically changing body motions. To experimentally verify the relative triboelectric polarity of InSe, additional control experiments were carried out by pairing InSe with different triboelectric materials under identical testing conditions [65, 66, 67]. As shown in Figure 2d–f, three representative contact pairs, including PET@InSe, PTFE@InSe, and PTFE@PET, were compared in terms of output voltage, current, and transferred charge. The output magnitude follows the order of PTFE@PET > PTFE@InSe > PET@InSe, indicating different triboelectric polarity differences among these material pairs. Since PTFE is a typical electronegative material and PET is relatively more tribopositive than PTFE, the intermediate output of PTFE@InSe and the weaker output of PET@InSe suggest that InSe is located between PET and PTFE in the relative triboelectric sequence under the present testing conditions. Therefore, InSe can be assigned as a relatively tribopositive material when paired with PTFE, which supports the charge‐distribution model of the PTFE/InSe‐based IS‐TENG. Figure 2g shows the *V*_OC_ output of the IS‐TENG as a function of time. Periodic voltage pulses with good regularity can be clearly observed, corresponding to successive contact‐separation cycles. The peak *V*_OC_ reaches approximately 773 V, indicating the strong charge generation capability of the PTFE/InSe triboelectric pair. The stable and repeated waveform further demonstrates the reliable electrical response of the fabricated device under cyclic mechanical excitation. As shown in Figure 2h, alternating positive and negative current peaks are observed during the repeated operation process, reflecting the bidirectional electron flow associated with the separation and recontact stages. The maximum *I*_SC_ reaches about 35 μA, confirming that the device is capable of generating a measurable current response with good repeatability. Figure 2i shows the *Q*_SC_ output of the IS‐TENG. A periodic charge accumulation and release process can be observed over time, with a maximum transferred charge of approximately 229 nC. The well‐defined cyclic profile indicates efficient *Q*_SC_ between the two electrodes during each working period. This result provides further evidence of the stable triboelectric output behavior of the device and supports its potential application in self‐powered energy harvesting and sensing systems. The high output performance of the IS‐TENG can be understood from the correlation between InSe morphology, interfacial contact, and charge transfer behavior. The compacted InSe powder layer exhibits a rough and layered/flaky morphology, which increases the effective contact area with the PTFE surface during repeated contact‐separation cycles. This enlarged interface facilitates triboelectric charge generation, while the relative polarity difference between PTFE and InSe provides the driving force for charge separation and electron transfer through the external circuit. Moreover, the increased output under higher applied force and larger separation distance further supports this mechanism, because stronger force improves interfacial contact and larger displacement enhances electrostatic induction. Therefore, the electrical performance of the IS‐TENG is attributed to the combined effects of the rough InSe contact morphology, PTFE/InSe triboelectric polarity difference, and effective charge‐transfer process.

### 3.3 Output Power, Energy Storage Behavior, and Practical Application of the IS‐TENG

**FIGURE 3:**
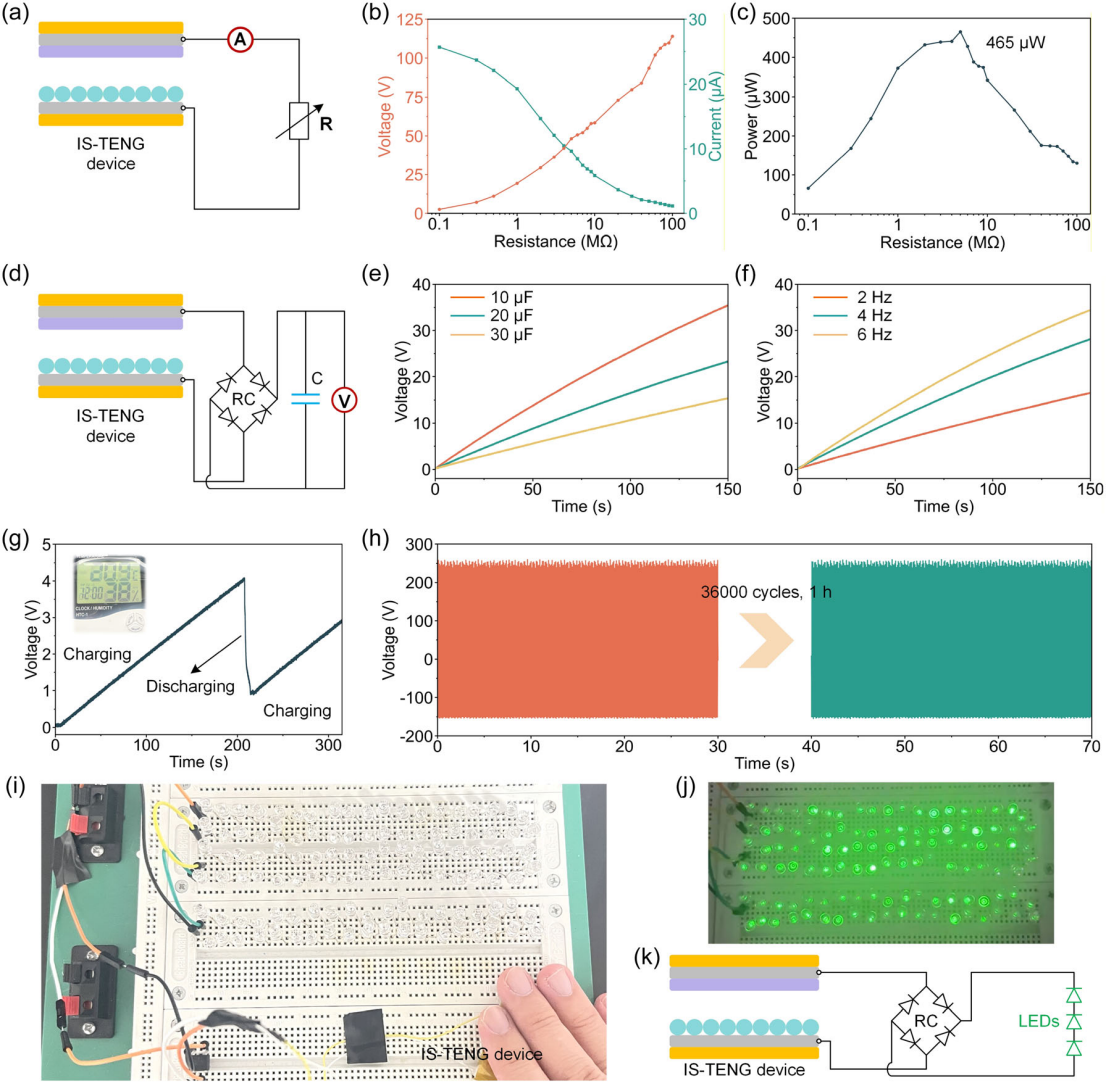
Energy harvesting capability, capacitor charging performance, durability, and practical powering demonstration of the IS‐TENG. (a) Schematic illustration of the external load measurement circuit for evaluating the electrical output characteristics of the IS‐TENG. (b) Variation of the output voltage and current with increasing load resistance. (c) Dependence of the output power on load resistance. (d) Circuit configuration used for rectification and capacitor charging. (e) Voltage–time charging curves of capacitors with different capacitances. (f) Effect of operating frequency on the capacitor charging behavior of the IS‐TENG. (g) Representative charging and discharging profile in an energy storage process. (h) Long‐term operational stability of the IS‐TENG after 36,000 cycles. (i) Photograph of the IS‐TENG integrated with an external LED circuit on a breadboard. (j) Photograph showing the illumination of green LEDs powered by the IS‐TENG. (k) Schematic circuit diagram of the LED demonstration system.

To assess the power‐delivery capability of the IS‐TENG, its electrical response was measured under a series of external resistive loads, as illustrated in Figure 3a. With the load resistance increasing from 0.1 to 100 MΩ, the output voltage rose progressively, while the current showed the opposite tendency. This behavior reflects the typical load‐dependent output feature of triboelectric generators, in which low‐resistance conditions favor current output and high‐resistance conditions favor voltage build‐up. Based on the corresponding voltage–current relationship, the output power was further calculated, as shown in Figure 3c. The power first increased with load resistance, reaching a maximum value of about 465 μW, corresponding to an output power density of 77.5 μW cm^−2^. The existence of this peak confirms that the IS‐TENG exhibits an appropriate impedance‐matching point and is capable of delivering useful electrical power to external loads. The energy‐storage capability of the IS‐TENG was further investigated through rectified capacitor‐charging experiments. As presented in Figure 3d, the alternating electrical output generated by the device was converted into a unidirectional signal through a rectifier and then used to charge capacitors, while the charging voltage was monitored in real time. Figure 3e compares the charging profiles of capacitors with different capacitances (10, 20, and 30 μF). Under identical operating conditions, all capacitors showed continuous voltage accumulation, demonstrating that the IS‐TENG can effectively transfer harvested energy into common storage units. A smaller capacitance led to a faster voltage rise, whereas larger capacitors required a longer charging duration because of their higher charge‐storage demand. The influence of excitation frequency on charging performance is displayed in Figure 3f. When the operating frequency was increased from 2 to 4 and 6 Hz, the charging process became more rapid, and the highest charging voltage was obtained at 6 Hz. These results indicate that increasing the mechanical excitation frequency can enhance charge‐transfer events and improve the energy‐storage efficiency of the IS‐TENG. To verify its practical usability, the IS‐TENG was also examined in a dynamic charging–discharging process and in direct powering demonstrations. As shown in Figure 3g, the capacitor voltage increased steadily during charging and dropped during discharge when the stored energy was consumed by an external device, confirming that the harvested energy could be stored and subsequently released in a controllable manner. It should be noted that the InSe layer was prepared by mechanically pressing powders onto the aluminum electrode, and the stable voltage output after 36,000 cycles suggests that no severe powder detachment or catastrophic interfacial degradation occurred during the tested period, as shown in Figure 3h. Quantitatively, by comparing the voltage amplitude after 36,000 cycles with the initial output amplitude, the voltage retention was calculated to be 100%, indicating that the IS‐TENG maintained its initial electrical output during the durability test. However, the long‐term adhesion and wear resistance of the pressed powder layer under more complex wearable conditions still require further systematic evaluation. In future work, microscopic morphology comparison before and after extended cycling, mass‐loss analysis, tape‐peeling tests, and abrasion‐resistance measurements will be carried out to further clarify the mechanical robustness of the InSe powder layer. For application‐oriented verification, the IS‐TENG was integrated with a simple external circuit on a breadboard, as shown in Figure 3i, and successfully used to illuminate 100 high‐power green LEDs with a working voltage of approximately 3.4 V, as displayed in Figure 3j.

### 3.4 Effect of Operating Parameters on the Electrical Output Characteristics of the IS‐TENG

**FIGURE 4:**
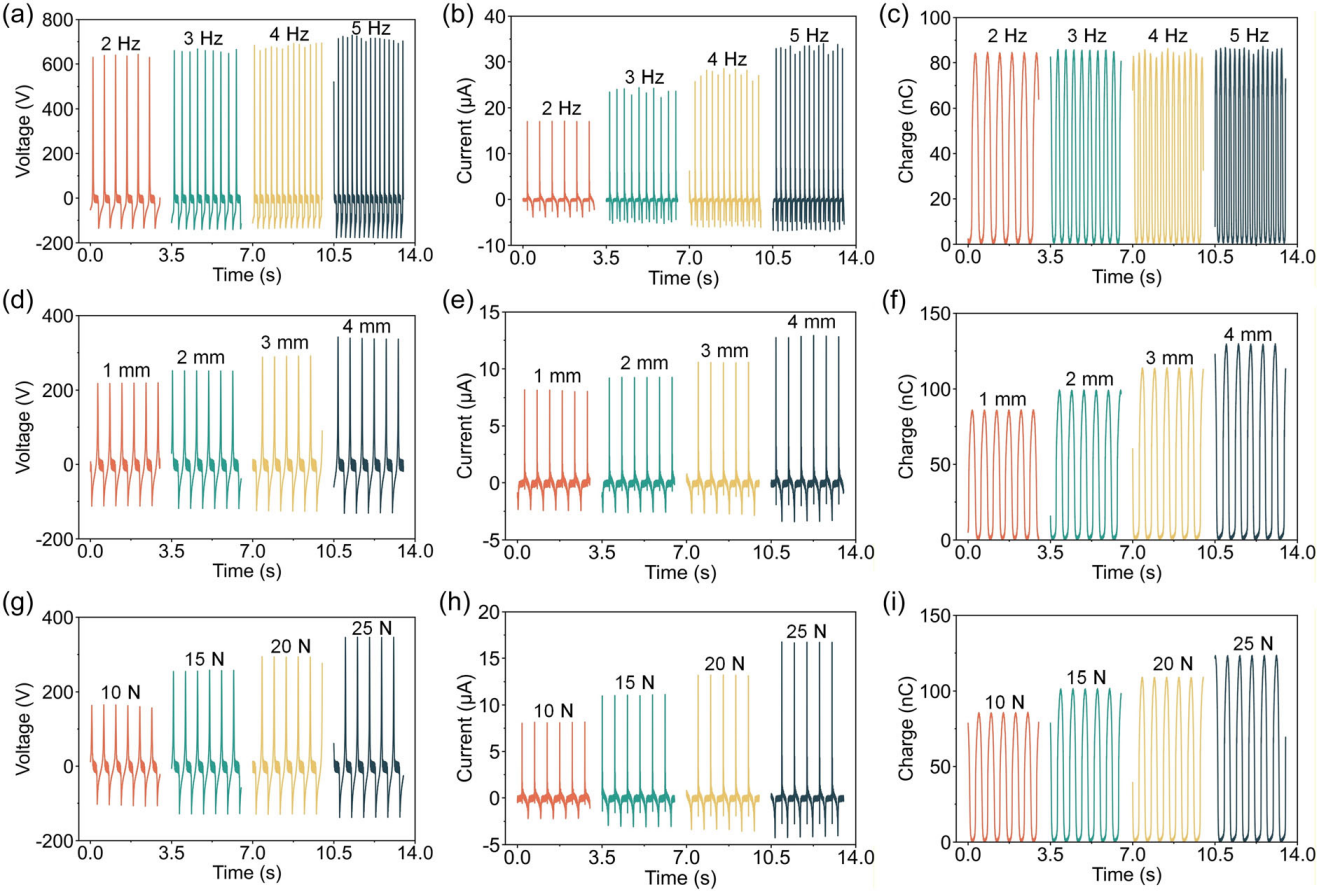
Dependence of the electrical output performance of the IS‐TENG on operating frequency, separation distance, and applied force. (a–c) *V*_OC_, *I*_SC_, and *Q*_SC_ of the IS‐TENG measured under different operating frequencies (2–5 Hz), respectively. (d–f) *V*_OC_, *I*_SC_, and *Q*_SC_ of the IS‐TENG measured under different separation distances (1–4 mm), respectively. (g–i) *V*_OC_, *I*_SC_, and *Q*_SC_ of the IS‐TENG measured under different applied forces (10–25 N), respectively.

Figure 4a presents the open‐circuit voltage of the IS‐TENG recorded at different operating frequencies from 2 to 5 Hz. Under each test condition, clear and periodic voltage pulses are generated, reflecting the repeated contact and separation between the triboelectric layers during cyclic mechanical excitation. With the increase in excitation frequency, the voltage peak gradually becomes higher, suggesting that more rapid actuation contributes to a stronger dynamic output response. At the same time, the interval between adjacent pulses is reduced as the frequency rises, which matches the shortened duration of each working cycle. These observations indicate that the open‐circuit voltage of the IS‐TENG is closely related to the actuation frequency and can be adjusted effectively by tuning the operating speed. Figure 4b shows the corresponding short‐circuit current obtained under the same frequency range. In comparison with the voltage signal, the current response exhibits a more pronounced increase as the frequency is raised. The peak current continuously grows from 2 to 5 Hz, implying that a higher working frequency promotes more rapid charge transport within a fixed time interval. Because short‐circuit current is directly associated with the velocity of charge migration in the external circuit, this tendency is fully consistent with the basic output behavior of TENGs. In addition, the highly regular current waveforms confirm that the IS‐TENG can operate steadily under different dynamic excitation conditions. Figure 4c displays the transferred charge measured at various excitation frequencies. Unlike the obvious changes observed in voltage and current, the transferred charge remains nearly unchanged across the tested frequency range, with only minor fluctuations in peak value. This finding indicates that the total charge produced in a single contact‐separation event is not strongly influenced by frequency itself. Instead, changing the frequency mainly alters how quickly the charge is delivered rather than how much charge is generated per cycle. Such a response is well aligned with the intrinsic working principle of contact‐separation mode TENG devices. As shown in Figure 4d, when the separation distance is increased from 1 to 4 mm, the open‐circuit voltage rises progressively. This enhancement can be explained by the larger electrostatic potential difference established between the two electrodes when the triboelectric layers move farther apart after contact. Therefore, a greater displacement in the contact‐separation process is beneficial for strengthening the voltage response of the device. Figure 4e presents the short‐circuit current under different separation distances. A trend similar to that of the voltage can be clearly observed, namely, that the current peak gradually increases as the displacement becomes larger. The enlarged separation gap enhances the electrostatic induction process and drives a more apparent electron flow through the external circuit, thereby improving the current output. Moreover, the pulse profiles remain stable and well defined at every tested displacement, further demonstrating the reproducibility and robustness of the generated electrical signals. Figure 4f shows the transferred charge as a function of separation distance. The amount of transferred charge increases continuously from 1 to 4 mm, indicating that a wider separation distance enables more induced charges to participate in the transfer process during each mechanical cycle. This result confirms that displacement is a key parameter governing the effective electrical output of the IS‐TENG, and it plays an essential role in enhancing and optimizing the device for mechanical energy conversion. Figure 4g displays the open‐circuit voltage measured under applied forces ranging from 10 to 25 N. As the force is gradually increased, the voltage peak also rises accordingly. This behavior can be attributed to the improved interfacial contact between the PTFE film and the InSe layer under stronger compression, which is favorable for generating a higher surface charge density during the triboelectrification process. Consequently, the enhanced voltage output reflects the strengthened interfacial interaction produced by greater mechanical loading. Figure 4h presents the corresponding short‐circuit current at different force levels. Similar to the voltage response, the current amplitude also increases steadily with increasing force. Stronger pressing force can improve the contact effectiveness between the friction surfaces, resulting in more efficient charge generation and a larger electron transfer during the subsequent release and recontact stages. The current pulses remain regular and repeatable throughout the tested force range, indicating that the IS‐TENG preserves stable output characteristics even when the external force varies. Figure 4i shows the transferred charge under different applied forces. The peak charge exhibits a clear upward trend as the force increases from 10 to 25 N, demonstrating that stronger mechanical input can generate a greater amount of triboelectric charge at the interface. This result further verifies that applied force is one of the most important factors influencing the electrical output of the device. Taken together, the results demonstrate that the IS‐TENG can be effectively regulated by external force, which not only benefits output enhancement for energy harvesting but also highlights its promise in self‐powered force sensing and related mechanical monitoring applications.

### 3.5 Effect of Environmental Conditions and Storage Time on the Electrical Output Performance of the IS‐TENG

**FIGURE 5:**
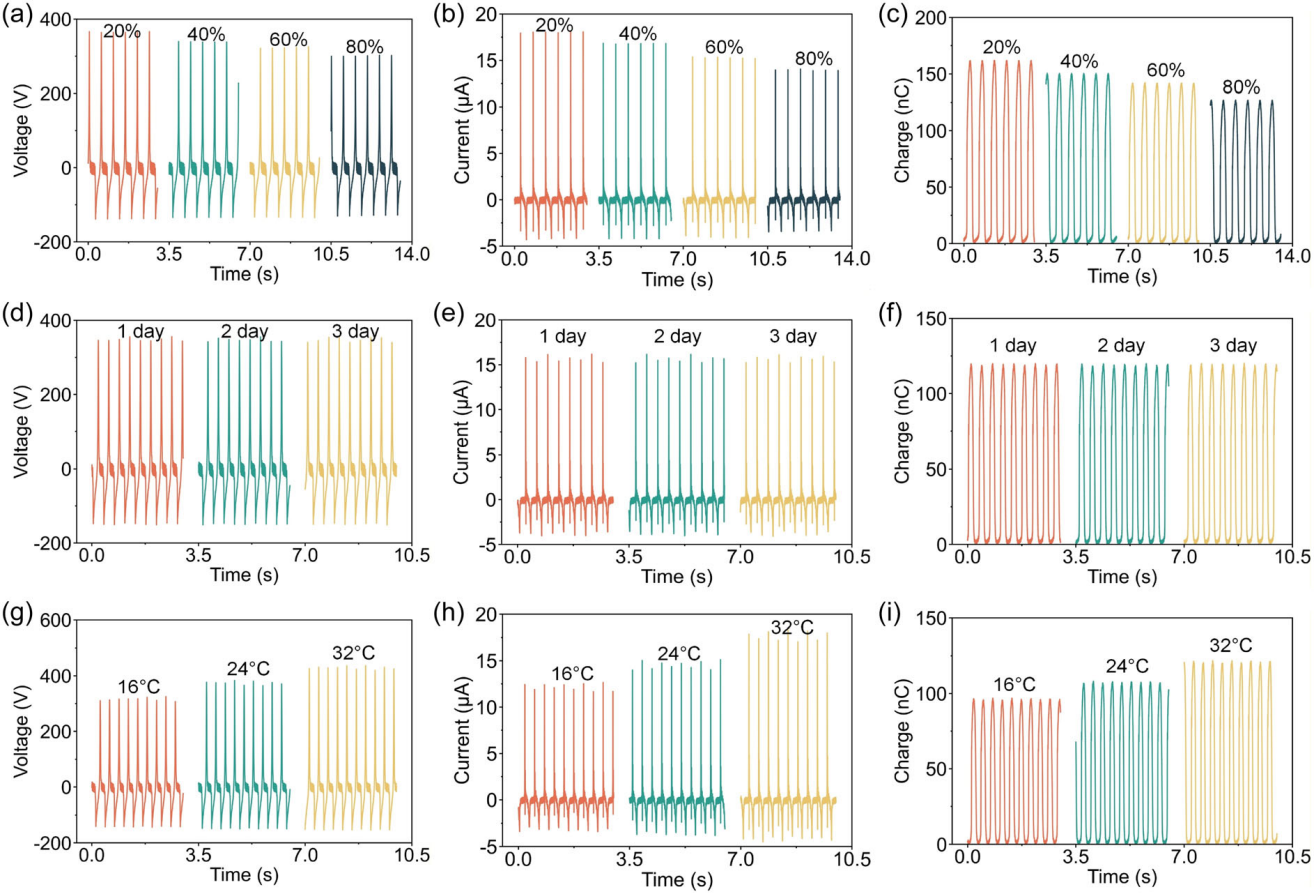
Dependence of the electrical output performance of the IS‐TENG on relative humidity, storage duration, and ambient temperature. (a–c) *V*_OC_, *I*_SC_, and *Q*_SC_ of the IS‐TENG measured under different relative humidity conditions (20%–80%), respectively. (d–f) *V*_OC_, *I*_SC_, and *Q*_SC_ of the IS‐TENG measured after different storage durations (1–3 days), respectively. (g–i) *V*_OC_, *I*_SC_, and *Q*_SC_ of the IS‐TENG measured at different ambient temperatures (16–32 °C), respectively.

Figure 5a presents the open‐circuit voltage *V*_OC_ of the IS‐TENG measured at different relative humidity levels of 20%, 40%, 60%, and 80%. Under all tested humidity conditions, the device generates clear and periodic voltage waveforms, indicating that the contact‐separation process remains functional throughout the entire humidity range. However, as the relative humidity increases, the voltage peak gradually declines. This tendency indicates that moisture in the ambient environment adversely affects the effective triboelectric output. A likely reason is that water molecules adsorbed on the triboelectric surfaces provide pathways for charge leakage, thereby weakening charge accumulation and reducing surface charge retention. Even so, the IS‐TENG still produces an obvious voltage response at 80% humidity, suggesting that the device retains a reasonable level of environmental tolerance under relatively humid conditions. Figure 5b shows the corresponding short‐circuit current *I*_SC_ obtained under the same humidity levels. A similar variation tendency can be observed, namely, that the current output decreases progressively as the relative humidity rises from 20% to 80%. Despite this reduction in magnitude, the current pulses remain periodic and clearly distinguishable, confirming that the electrical response of the IS‐TENG is still stable in humid environments. The gradual decrease in current is consistent with the reduction in effective triboelectric charge density caused by environmental moisture, further demonstrating that humidity is a key external factor influencing device output. Figure 5c displays the *Q*_SC_ under different humidity conditions. In line with the voltage and current results, the transferred charge also shows an overall decreasing trend with increasing humidity. This observation suggests that the total amount of effective charge participating in each contact‐separation cycle is reduced under high‐humidity conditions. The humidity‐dependent decrease in *V*_OC_, *I*_SC_, and *Q*_SC_ can be mainly attributed to moisture adsorption on the triboelectric surfaces. Water molecules may form a thin conductive layer at the interface, which accelerates surface charge dissipation, weakens charge retention, and partially screens the electrostatic induction process, thereby reducing the effective triboelectric output. For practical wearable applications under real environmental conditions, protection strategies such as flexible hydrophobic encapsulation, edge sealing, waterproof yet deformable packaging, and moisture‐resistant fluoropolymer coatings could be adopted to reduce water adsorption while maintaining the contact‐separation operation of the device. These strategies would be helpful for improving the long‐term stability of the IS‐TENG under humid air, sweat exposure, and daily wearable conditions. Figure 5d presents the *V*_OC_ measured after storage for 1, 2, and 3 days. The recorded voltage profiles after these different storage periods remain highly comparable in both waveform shape and output amplitude, with no apparent attenuation observed. This result indicates that the PTFE/InSe triboelectric structure maintains its functional state well during short‐term storage and is capable of preserving a stable voltage response over time. Figure 5e shows the *I*_SC_ measured after the same storage durations. The peak current values remain almost unchanged among the three tested conditions, and the current waveforms continue to exhibit regular periodicity. Such behavior demonstrates that the IS‐TENG experiences little variation in current output after being stored for several days, implying good reproducibility of its electrical response and satisfactory structural reliability of the fabricated device. Figure 5f gives the *Q*_SC_ output after storage for 1, 2, and 3 days. Similar to the voltage and current results, the charge peaks remain nearly constant, with only very small fluctuations. The stable charge response further supports the conclusion that short‐term storage does not introduce noticeable deterioration in triboelectric performance. Taken together, the results show that the IS‐TENG possesses good short‐term storage stability and can maintain dependable output behavior after temporary storage before practical use. As shown in Figure 5g, the *V*_OC_ was measured at 16, 24, and 32 °C. The voltage amplitude increases gradually as the temperature rises, while the signal pattern remains periodic and stable at each temperature. This result indicates that a moderate increase in ambient temperature is beneficial to the triboelectric performance of the device. Such an improvement may originate from temperature‐related changes in interfacial contact condition, charge excitation, or charge retention behavior during repeated contact‐separation motion. Figure 5h presents the *I*_SC_ under the same temperature conditions. The current output follows a trend similar to that of the voltage, showing a gradual increase from 16 to 32 °C. Since the *I*_SC_ is highly sensitive to the charge transfer rate, the enhanced current response implies that higher temperature facilitates more effective charge generation and transport during cyclic operation. In addition, the well‐maintained pulse regularity confirms the reliability of the electrical output under different thermal environments. Figure 5i shows the *Q*_SC_ measured at 16, 24, and 32 °C. The charge peak also increases with increasing temperature, which agrees well with the variation trends observed for voltage and current in Figure 5g,h. This finding demonstrates that, within the investigated temperature range, ambient temperature contributes positively to the triboelectric output of the IS‐TENG. Hence, the results indicate that environmental factors exert different influences on the output behavior of the IS‐TENG. Increased humidity tends to weaken the device performance by reducing the effective charge generation and transfer capability, whereas a moderate rise in temperature is favorable for enhancing the electrical response. In contrast, short‐term storage produces almost no noticeable influence on voltage, current, or transferred charge. These observations demonstrate that the IS‐TENG not only exhibits acceptable tolerance to environmental variation but also maintains stable performance after storage, which supports its potential for practical applications under real operating conditions.

### 3.6 Wearable Application of the IS‐TENG for Human Joint Posture Monitoring and Motion Sensing

**FIGURE 6:**
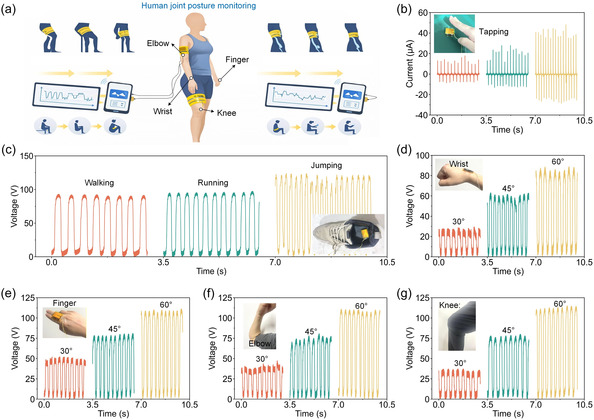
Human motion sensing and joint posture monitoring using the wearable IS‐TENG. (a) Schematic diagram of the wearable application of the IS‐TENG for monitoring joint motion at different body locations. (b) Current output of the IS‐TENG under finger tapping. (c) Voltage output of the IS‐TENG under different motion states, including walking, running, and jumping. (d–g) Voltage responses of the IS‐TENG attached to the wrist, finger, elbow, and knee, respectively, under different bending angles (30°, 45°, and 60°).

Figure 6a depicts a representative wearable‐use framework of the IS‐TENG for monitoring human joint motion and body posture. As illustrated in the schematic, the device can be positioned on several articulated regions of the body, such as the elbow, wrist, finger, and knee, where deformation generated during bending or extension is transformed into measurable electrical signals. These signals can then be transmitted to an external readout interface or portable electronic terminal for immediate waveform display and subsequent interpretation of motion states. The posture symbols and joint‐bending sketches shown in the figure further suggest that the IS‐TENG is capable of distinguishing multiple forms of body movement and posture variation. This application model demonstrates that the device is suitable for wearable, battery‐free biomechanical monitoring and provides a practical basis for motion recognition, posture evaluation, and related intelligent sensing tasks. Figure 6b presents the current response of the IS‐TENG during repeated finger‐tapping operation. During intermittent manual tapping, distinct alternating current pulses are continuously generated, indicating that the device can respond rapidly and sensitively to transient mechanical input. The output signals remain readily recognizable over consecutive tapping cycles, and no obvious signal disorder or severe attenuation is observed, which confirms the stable dynamic response of the device under repeated touch stimulation. The inset photograph gives direct visual evidence of the manual triggering process, showing the sensor being actuated by a finger in a practical testing condition. These results confirm that the IS‐TENG can be used for real‐time touch‐event recording and human‐interactive signal collection, which is important for wearable tactile interfaces and self‐powered trigger sensing. Figure 6c shows the voltage output of the IS‐TENG under three typical motion conditions, including walking, running, and jumping. In the walking state, the device produces periodic voltage waveforms with relatively uniform amplitude and moderate pulse frequency, corresponding to the regular rhythm and comparatively mild impact generated during normal gait. When the subject changes to running, the output pulses become more closely spaced and the signal intensity increases, reflecting the higher step frequency and stronger mechanical excitation associated with faster locomotion. Under jumping conditions, the voltage exhibits the largest amplitude and the most obvious fluctuation among the three tested activity modes, which can reasonably be attributed to the stronger impact load and more abrupt body movement involved in jumping. The inset image shows the IS‐TENG integrated with footwear, providing direct evidence that the device can be conveniently incorporated into wearable systems for practical motion acquisition. These results demonstrate that different exercise patterns can be differentiated through their characteristic electrical signatures, indicating the feasibility of the IS‐TENG for exercise‐state recognition and wearable activity analysis. Figure 6d displays the voltage output of the IS‐TENG mounted on the wrist at bending angles of 30°, 45°, and 60°. When the wrist bends by 30°, the recorded voltage remains relatively small, suggesting that the deformation imposed on the device is still limited at this angle. As the bending angle increases to 45°, the voltage response becomes clearly stronger, indicating that larger mechanical strain is imposed on the sensor. When the wrist reaches 60°, the output signal attains the highest amplitude among the three conditions, reflecting the most pronounced deformation‐induced triboelectric response. The inset photograph provides a direct image of the sensor attached to the wrist during testing. These observations demonstrate that the IS‐TENG can sensitively follow wrist articulation and can translate different bending states into distinguishable electrical outputs, thereby offering a simple route for wearable wrist‐motion tracking. Figure 6e presents the voltage response of the IS‐TENG attached to the finger under bending angles of 30°, 45°, and 60°. A trend similar to that observed for the wrist can be identified, namely, that the output signal gradually increases as the finger bending angle becomes larger. The voltage produced at 30° is comparatively weak, whereas the signal becomes much more obvious at 45°, and the highest output is achieved at 60°. The inset photograph shows the placement of the device on the finger during the measurement process. These results indicate that the IS‐TENG can operate effectively as a self‐powered finger‐motion sensor and can provide clear electrical differentiation among distinct finger postures. Such an ability is valuable for wearable gesture capture, finger‐action recognition, and other fine‐motion monitoring applications. Figure 6f shows the voltage output of the IS‐TENG when it is fixed on the elbow and tested under bending angles of 30°, 45°, and 60°. As the bending degree increases, the output amplitude rises correspondingly, showing the same overall tendency as in the wrist and finger measurements. Compared with small‐angle bending, larger elbow motion produces stronger deformation of the attached device and therefore leads to a more intense triboelectric response. The inset image gives a practical view of the IS‐TENG attached to the elbow region. This result confirms that the device can reliably monitor elbow movement and respond to angular variation in an obvious and repeatable manner, further supporting its applicability in wearable posture sensing and upper‐limb motion analysis. Figure 6g presents the voltage output of the IS‐TENG used for knee‐joint monitoring at bending angles of 30°, 45°, and 60°. With increasing knee flexion, the voltage signal becomes progressively stronger, and the output amplitude clearly rises from the 30° condition to the 60° condition. Because the knee usually undergoes relatively large‐scale mechanical motion during body activity, the sensor attached to this region experiences more substantial deformation, which gives rise to a more pronounced electrical response. The inset photograph shows the sensor fixed on the knee during the monitoring process. These results demonstrate that the IS‐TENG can function effectively as a self‐powered device for lower‐limb posture tracking and dynamic knee‐angle sensing, which is of particular interest for wearable rehabilitation monitoring, sports‐motion evaluation, and gait‐related studies. Overall, the results presented in Figure 6 demonstrate that the IS‐TENG responds well to both short‐duration tactile triggering and continuous joint deformation. The device can not only discriminate among different whole‐body activity modes but also resolve changes in bending angle at multiple body locations, including the wrist, finger, elbow, and knee. Such performance highlights the strong potential of the IS‐TENG in wearable electronics, posture assessment, exercise‐state recognition, and self‐powered biomechanical monitoring systems. It should be noted that the wearable tests are intended as proof‐of‐concept demonstrations to verify the feasibility of the IS‐TENG for self‐powered human motion sensing. Because these demonstrations were performed under real human motion conditions, the applied force, contact speed, bending trajectory, and wearing position were not strictly controlled, making it difficult to accurately extract quantitative sensing parameters such as sensitivity, response time, detection limit, signal‐to‐noise ratio, and interuser reproducibility from the present data. Future work will therefore focus on systematic sensor calibration using standardized mechanical actuation and multiuser testing protocols, including force/angle‐dependent sensitivity, response/recovery time, minimum detectable motion, signal‐to‐noise ratio, and long‐term reproducibility under practical wearable conditions. It should be noted that the present human motion tests were conducted on a single volunteer and were intended to demonstrate the feasibility of the IS‐TENG for wearable self‐powered motion sensing. Future studies will include multisubject experiments, standardized motion protocols, and statistical analysis to further evaluate interuser reproducibility and real‐world wearable reliability.

## 4 Conclusions

In summary, an IS‐TENG was successfully developed through a simple and scalable layer‐by‐layer assembly strategy for biomechanical energy harvesting and self‐powered human motion monitoring. By employing Kapton as the flexible substrate, aluminum foil as the conductive electrode, PTFE as one triboelectric layer, and compacted InSe powders as the counterpart friction material, the fabricated IS‐TENG exhibited excellent electrical output performance, delivering a high *V*_OC_ of 773 V, an *I*_SC_ of 35 μA, a *Q*_SC_ of 229 nC, and a maximum output power of 465 μW. The device also demonstrated effective capacitor‐charging capability, good long‐term durability over 36,000 operating cycles, and stable output responses under different frequencies, separation distances, applied forces, humidity levels, temperatures, and storage durations. More importantly, the IS‐TENG could be directly applied as a wearable self‐powered sensor for finger tapping detection, discrimination of walking, running, and jumping states, and monitoring of joint motions at different bending angles for the wrist, finger, elbow, and knee. These results confirm that the proposed IS‐TENG not only serves as an efficient energy harvester but also offers a promising strategy for constructing flexible, lightweight, and low‐power wearable sensing platforms based on functional materials. This work provides useful insight into the development of self‐powered motion‐monitoring systems for wearable electronics, intelligent healthcare, and related human–machine interaction applications. Nevertheless, the present IS‐TENG still has several limitations that should be addressed in future studies. Although stable output was demonstrated under controlled humidity, temperature, storage, and cycling conditions, its long‐term environmental stability under real wearable scenarios, such as prolonged sweat exposure, repeated body deformation, and complex daily‐motion interference, still requires further evaluation. Future work will focus on improving device encapsulation, mechanical robustness, and signal stability, as well as integrating the IS‐TENG with compact signal‐processing circuits, wireless transmission modules, and flexible wearable platforms to promote its practical application in real‐world self‐powered motion monitoring and human–machine interaction systems.

## Funding

This study was supported by Basic and Applied Basic Research Foundation of Guangdong Province (2023A1515110377).

## Conflicts of Interest

The authors declare no conflicts of interest.

## Data Availability

Data are available on request from the authors.
